# Characterization of patients transported with extracorporeal respiratory and/or cardiovascular support in the State of São Paulo, Brazil

**DOI:** 10.5935/0103-507X.20180052

**Published:** 2018

**Authors:** Ho Yeh Li, Pedro Vitale Mendes, Livia Maria Garcia Melro, Daniel Joelsons, Bruno Adler Maccagnan Pinheiro Besen, Eduardo Leite Viera Costa, Adriana Sayuri Hirota, Edzangela Vasconcelos Santos Barbosa, Flavia Krepel Foronda, Luciano Cesar Pontes Azevedo, Thiago Gomes Romano, Marcelo Park

**Affiliations:** 1 Hospital das Clínicas, Faculdade de Medicina, Universidade de São Paulo - São Paulo (SP), Brasil.; 2 Hospital TotalCor - São Paulo (SP), Brasil.; 3 Hospital Sírio Libanês - São Paulo (SP), Brasil.; 4 Unidade de Terapia Intensiva Oncológica, Hospital São Luiz, Rede D'Or - São Paulo (SP), Brasil.

**Keywords:** Artificial, respiration, Respiratory insufficiency, Extracorporeal membrane oxygenation, Transportation of patients, Critical illness, Intensive care units

## Abstract

**Objective:**

To characterize the transport of severely ill patients with extracorporeal respiratory or cardiovascular support.

**Methods:**

A series of 18 patients in the state of São Paulo, Brazil is described. All patients were consecutively evaluated by a multidisciplinary team at the hospital of origin. The patients were rescued, and extracorporeal membrane oxygenation support was provided on site. The patients were then transported to referral hospitals for extracorporeal membrane oxygenation support. Data were retrieved from a prospectively collected database.

**Results:**

From 2011 to 2017, 18 patients aged 29 (25 - 31) years with a SAPS 3 of 84 (68 - 92) and main primary diagnosis of leptospirosis and influenza A (H1N1) virus were transported to three referral hospitals in São Paulo. A median distance of 39 (15 - 82) km was traveled on each rescue mission during a period of 360 (308 - 431) min. A median of one (0 - 2) nurse, three (2 - 3) physicians, and one (0 - 1) physical therapist was present per rescue. Seventeen rescues were made by ambulance, and one rescue was made by helicopter. The observed complications were interruption in the energy supply to the pump in two cases (11%) and oxygen saturation < 70% in two cases. Thirteen patients (72%) survived and were discharged from the hospital. Among the nonsurvivors, there were two cases of brain death, two cases of multiple organ dysfunction syndrome, and one case of irreversible pulmonary fibrosis.

**Conclusions:**

Transportation with extracorporeal support occurred without serious complications, and the hospital survival rate was high.

## INTRODUCTION

The use of extracorporeal membrane oxygenation (ECMO) support has increased in recent years,^(1)^ especially following the pandemic of influenza A (H1N1) virus pneumonitis.^(2-4)^ Although the results of previous randomized trials in which ECMO was used for respiratory support are inconclusive,^(5,6)^ new technologies^(7)^ associated with the application of ultraprotective mechanical ventilation^(8)^ have improved survival and the quality of life when ECMO is used for patients with severe respiratory failure.^(9,10)^

The high cost of the training and support required for ECMO use may have a negative economic impact, especially in developing countries.^(11)^ However, the high cost of the initial installation of the system is compensated for by its low cost of maintenance and the good outcomes obtained when ECMO support is used with adequate staff training, making this therapy cost-effective in developed countries^(9,12)^ and potentially cost-effective in developing countries.^(13)^

Considering that ensuring the availability of appropriate staff in health centers with a relatively small occupancy rate may increase the cost of extracorporeal support, ECMO-equipped transport to specialized centers has been made available at an acceptable cost, with high survival rates and improvement in the quality of life.^(4,9)^

Considering the importance of transport with ECMO, the objective of this study was to characterize the transport performed by our team in the State of São Paulo since 2011.

## METHODS

Data were retrieved from a prospectively collected database. The analysis of the database was assessed and approved by the Research Ethics Committee of the *Hospital das Clínicas* of the *Faculdade de Medicina* of the *Universidade de São Paulo* (USP) (number 107,443), and the requirement for informed consent was waived. Data on each patient were collected as previously described^(14,15)^ using an online worksheet in the REDCap system.^(16)^

The contact was made by telephone by a local team member. Data were stored in an online spreadsheet. The severity of the patient's condition was determined, and the indications and contraindications for extracorporeal support were analyzed. The indication and contraindication criteria were previously described by our group.^(17)^ These criteria were modified slightly because the initial results were suboptimal due to occasional problems in the initial experience.^(14,18-20)^ The current criteria are described in the Supplementary Material. Although the contraindication criteria were restrictive, special situations that generated doubts were discussed by our group. In cases in which the indication criteria were fulfilled or in which there were doubts, the remaining members of the team were contacted, and the final decision on whether or not to undertake support was made by the team as a whole.

The rescue team was composed of at least three professionals, of whom at least two were physicians (the third professional was a physician, a nurse, or a physical therapist). All the professionals who formed the team were trained to operate the system and to engage in open and direct communication with patients, relatives, and caregivers.

All the professionals made an initial assessment of the patients. When there was agreement about the indication, the two physicians were responsible for cannulation, and the third professional was responsible for communication with the patient's relatives and for equipment assembly, including priming the system.

Because an adequate transport system was not available, the requesting center was responsible for transporting the hospital staff to the requested location by ambulance or private transport. The team was responsible for carrying some equipment on the mission, including an ECMO system, a voltage stabilizer for the ECMO pump, two infusion pumps, a noninvasive blood pressure measurement system, and an oximeter. The transport of these items was confirmed using a checklist before departure for the mission. The remaining monitoring and support were provided by the ambulance in charge of the return transport.

Initial support, initial patient stabilization, and migration to protective/ultraprotective ventilation were performed in the presence of all three professionals. The stepwise technique used in this process was previously described.^(14)^ The ECMO system included a polymethylpentene membrane oxygenator connected to the following centrifugal pumps: (1) Rotaflow/Jostra Quadrox-D/Permanent Life Support (PLS; Maquet Cardiopulmonary AG, Hirrlingen, Germany), and (2) a BioPump with campanula and Affinity^TM^ circuit (Medtronic Inc, MN, USA) with a BIOCUBE 6000 membrane (Nipro Ltda, Sorocaba, São Paulo, Brazil).

Ambulances could be used to transport critical patients provided these vehicles had a mechanical ventilator capable of delivering at least 10cmH_2_O of positive end expiratory pressure and an inverter with a power of at least 2,000 watts. The latter feature was requested because less powerful inverters were not able to keep the ECMO pump working together with the other required devices. Team workload was reduced during transport by not carrying the ECMO thermoregulator and by carefully keeping the ambulance air conditioner off to avoid excessive cooling of the patient.

### Statistical analysis

The data were considered nonparametric because of the small sample size and are reported as the median [25^th^ - 75^th^ percentile] if quantitative and as the number of occurrences and percentages if qualitative. The comparisons between the groups presented in the tables were performed using the Mann-Whitney test for quantitative data and Fisher's exact test for qualitative data. The confidence interval of the survivor ratio was calculated according to the method described by the Association of Public Health Observatories^(21)^ using R software for calculations and graph creation.^(22)^

## RESULTS

The ECMO program was initiated in 2011, and the transport of ECMO patients began in the same year.^(14)^ A flowchart of the 28 requests for extracorporeal support outside the referral hospitals is shown in figure 1S. The first seven patients in this series were described in another publication.^(15)^ During the six years of the program, 18 patients in the state of São Paulo were rescued and transported with ECMO support by our team. Seventeen patients received exclusive respiratory support (veno-venous - VV configuration), and one patient received respiratory and cardiovascular support (veno-arterial - VA configuration). A profile of the patients is shown in table 1. The characteristics of the patients shortly before initiation of the support are shown in table 2. The Respiratory ECMO Survival Prediction Score (RESP score) and the tidal volume in pre-ECMO mechanical ventilation differed significantly in survivors and nonsurvivors. The data on the rescue missions and complications during transport are shown in table 3. The referral hospitals were *Hospital Sírio Libanês* (two patients), *Hospital TotalCor* (two patients), and the *Hospital das Clínicas* of São Paulo (14 patients).

**Table 1:** General characteristics of patients transported with extracorporeal membrane oxygenation support

Characteristics	All patients (n = 18)	Survivors (n = 13)	Nonsurvivors (N = 5)	p value*
Age (years)	29 [25 - 31]	28 [25 - 31]	29 [27 - 31]	0.621
Female	11 (61)	9 (69)	2 (40)	0.326
SAPS 3	84 [68 - 92]	84 [66 - 88]	80 [73 - 95]	0.961
Mortality predicted in South America (%)†	89 [66 - 94]	89 [62 - 92]	86 [76 - 95]	1.000
Mortality predicted in Europe (%)†	76 [46 - 85]	76 [42 - 81]	71 [58 - 88]	0.961
SOFA	13 [9 - 16]	14 [10 - 16]	13 [9 - 13]	0.584
Weight (kg)	74 [55 - 84]	72 [50 - 78]	84 [60 - 84]	0.298
Height (cm)	165 [160 - 185]	165 [160 - 170]	180 [160 - 185]	0.344
Associated clinical conditions				0.920
Systemic arterial hypertension	2 (11)	1 (8)	1 (20)	
Diabetes mellitus	1 (9)	1 (8)	0 (0)	
Gestational complications	1 (9)	1 (8)	0 (0)	
Postpartum complications	3 (17)	3 (38)	0 (0)	
Systemic lupus erythematosus	1 (9)	1 (8)	0 (0)	
HIV/AIDS	1 (9)	0 (0)	1 (20)	
Etiological diagnoses				0.810
Alveolar hemorrhage due to lupus	1 (9)	1 (8)	0 (0)	
Leptospirosis	3 (17)	3 (38)	0 (0)	
H3N2 Influenza A virus	1 (9)	0 (0)	1 (20)	
H1N1 Influenza A virus	3 (17)	2 (38)	1 (20)	
Influenza B virus	1 (9)	1 (8)	0 (0)	
Varicella zoster virus	1 (9)	1 (8)	0 (0)	
Respiratory syncytial virus	1 (9)	1 (8)	0 (0)	
Coronavirus	1 (9)	1 (8)	0 (0)	
Epstein-Barr virus	1 (9)	1 (8)	0 (0)	
Nosocomial pneumonia	1 (9)	1 (8)	0 (0)	
Aspiration pneumonia	1 (9)	0 (0)	1 (20)	
Necrotizing pneumonia	1 (9)	0 (0)	1 (20)	
Pelvic septic thrombophlebitis	1 (9)	1 (8)	0 (0)	
*Pneumocystis jirovecii*	1 (9)	0 (0)	1 (20)	

SAPS 3 - Simplified Acute Physiology Score 3; SOFA - Sequential Organ Failure Assessment Score.

* Value of comparison between survivors and nonsurvivors;

† calculation was performed using the logit of SAPS 3 for South America and Western Europe, respectively. The results are expressed as the median [interquartile 25 - 75] or the number (%).

**Table 2:** Respiratory and hemodynamic characteristics of patients who received extracorporeal membrane pre-oxygenation support

Characteristics	All patients (n = 18)	Survivors (n = 13)	Nonsurvivors (N = 5)	p value*
Murray score	3.7 [3.0 - 4.0]	3.5 [3.0 - 4.0]	3.8 [3.0-4.0]	0.448
Mechanical ventilation time (days)	7 [1 - 11]	5 [1 - 8]	8 [7-15]	0.113
ICU time (days)	7 [2 - 11]	5 [1 - 9]	8 [8-15]	0.199
RESP score	0.00 [-2.00 - 2.00]	1.50 [-0.25 - 3.25]	-2.00 [-3.00 - -1.00]	0.023
Survival rate (RESP) (%)	50 [40 - 60]	58 [48 - 69]	40 [35 - 45]	0.034
SAVE score	0.00	0.00	---	---
Survival rate (SAVE) (%)	40	40	---	---
Mechanical ventilation				
Pressure-controlled mode	14 (78)	10 (77)	4 (80)	1.000
Volume-controlled mode	4 (22)	3 (23)	1 (20)	
PEEP (cmH_2_O)	14 [10 - 18]	12 [10 - 15]	17 [13 - 18]	0.269
FiO_2_ (%)	100 [100 - 100]	100 [100 - 100]	100 [100 - 100]	0.288
Tidal volume (mL/kg)	4 [4 - 6]	5 [4 - 6]	3 [3 - 4]	< 0.001
Respiratory rate (ipm)	28 [25 - 35]	28 [25 - 35]	28 [28 - 35]	0.723
Plateau pressure (cmH_2_O)	33 [30 - 35]	34 [30 - 35]	32 [31 - 35]	0.960
Blood gas analysis				
pH	7.27 [7.08 - 7.35]	7.27 [7.09 - 7.35]	7.10 [7.00 - 7.34]	0.622
PaO_2_ (mmHg)	54 [38 - 60]	58 [39 - 65]	50 [45 - 60]	0.882
PaCO_2_ (mmHg)	61 [46 - 90]	53 [42 - 80]	90 [49 - 90]	0.429
SBE (mEq/L)	1.5 [-2.5 - 4.8]	1.0 [-3.0 - 6.0]	2.0 [0.0 - 4.0]	0.805
Lactate (mEq/L)	2.7 [2.2 - 3.9]	2.7 [2.2 - 3.3]	2.7 [2.7 - 4.4]	0.692
P/F ratio (mmHg)	55 [39 - 60]	60 [39 - 65]	50 [45 - 60]	0.657
Salvage therapy				
Alveolar recruitment	15 (84)	10 (77)	5.100	0.638
Nitric oxide	2 (11)	0 (0)	2 (40)	0.114
Prone position	12 (67)	7 (54)	5.100	0.193
Curarization	15 (84)	10 (77)	5.100	0.638
TGI	2 (11)	0 (0)	2 (40)	1.000
Corticosteroids	12 (67)	8 (62)	4 (40)	0.852
Hemodynamic support				
Noradrenaline	16 (89)	12 (92)	4 (80)	1.000
Vasopressin	4 (22)	1 (8)	3 (60)	0.078
Adrenaline	3 (17)	1 (8)	2 (40)	1.000
Dobutamine	3 (17)	3 (23)	0 (0)	0.638

ICU - intensive care unit; RESP score - Respiratory ECMO Survival Prediction Score; SAVE score - Survival After Veno-Arterial-ECMO Score; PEEP - positive end-expiratory pressure; FiO_2_ - fraction of inspired oxygen; PaO_2_ - partial pressure of oxygen; PaCO_2_ - partial pressure of carbon dioxide; SBE - standard base excess; P/F ratio - PaO_2_/FiO_2_ ratio; TGI - tracheal gas insufflation.

* Value of comparison between survivors and nonsurvivors. The results are expressed as the median [interquartile 25 - 75] or the number (%).

**Table 3:** Characteristics of missions and transportation

Characteristics	All patients (n = 18)	Survivors (n = 13)	Nonsurvivors (N = 5)	p value*
Hospital of origin				
Public	13 (72)	9 (69)	4 (80)	0.057
Private	5 (28)	4 (31)	1 (20)	
Referral hospital				
Public	14 (78)	10 (77)	4 (80)	1.000
Private	4 (22)	3 (23)	1 (20)	
Mission time (minutes)	360 [308 - 431]	360 [300 - 420]	420 [345 - 435]	0.520
Distance traveled (km)	39 [15 - 82]	40 [12 - 90]	37 [23 - 40]	1.000
Professionals involved				
Nurses (number per mission)	1 [0 - 2]	1 [0 - 2]	1 [1 - 2]	0.675
Doctors (number per mission)	3 [2 - 3]	3 [2 - 3]	2 [2 - 3]	0.437
Physical therapists (number per mission)	1 [0 - 1]	1 [0 - 1]	1 [0 - 1]	1.000
Transportation vehicle				
Ambulance	17 (94)	12 (92)	5.100	1.000
Helicopter	1 (6)	1 (8)	0 (0)	
Complications				
Insufficient power	2 (11)	1 (8)	1 (20)	1.000
SpO_2_ < 70%	2 (11)	2 (16)	0 (0)	0.890
SpO_2_ < 85%	4 (22)	2 (16)	2 (40)	0.900
Hemodynamic worsening	0 (0)	0 (0)	0 (0)	1.000
Temperature < 35 ºC	1 (6)	1 (8)	0 (0)	0.900

SpO_2_ - blood oxygen saturation.

* Value of comparison between survivors and nonsurvivors. The results are expressed as the number (%) or the median [interquartile 25 - 75].

The data on the extracorporeal support are shown in table 4. Respiratory support was provided using the femoral-jugular configuration, and veno-arterial support (one case) was provided using the femoral-femoral configuration. The venous cannulae were 21 - 22 Fr, and the arterial cannulae were 16 - 19 Fr. Apart from veno-arterial cannulation, anticoagulation was started upon patient arrival at the referral hospital. Five patients did not use anticoagulation at any time because of pulmonary hemorrhage (four cases) or the presence of cerebral vasculitis with hemorrhagic areas (one case). None of the evaluated patients had a change of itinerary or a change in the support configuration related to initial cannulation. The final results are shown in table 5. The minimum and maximum duration of support was 3 and 60 days, respectively. Of the 18 patients, 13 (72%, 95%CI 49 - 88) survived to hospital admission (Figure 2S). Of the survivors, only one patient needed dialysis after hospital admission, and none required home oxygen therapy. The individual patient data are presented in table 1S.

**Table 4:** Support and complications of patients in the intensive care unit

Characteristics	All patients (n = 18)	Survivors (n = 13)	Nonsurvivors (N = 5)	p value*
Cannulation				
Veno-venous ECMO	17 (94)	12 (92)	5.100	1.000
Veno-arterial ECMO	1 (6)	1 (8)	0 (0)	
Initial settings for ECMO				
Blood flow (mL/min)	4725 [4.300 - 5.498]	4500 [4.300 - 5.400]	5000 [4.300 - 6.400]	0.521
Sweep flow (L/min)	6.0 [4.0 - 8.0]	4,5 [4.0 - 8.0]	6.0 [6.0 - 10.0]	0.136
FiO_2_ (%)	100 [100 - 100]	100 [100 - 100]	100 [100 - 100]	1.000
Initial fan settings				
PSV mode	3 (17)	3 (23)	0 (0)	0.372
PCV mode	14 (78)	9 (69)	5.100	
APRV mode	1 (6)	1 (8)	0 (0)	
Tidal volume (mL/kg)	2.1 [0.89 - 2.71]	2.33 [0.80 - 2.93]	0.95 [0.91 - 2.20]	0.460
PEEP (cmH2O)	10 [10 - 10]	10 [10 - 10]	10 [10 - 12]	0.105
Plateau pressure (cmH_2_O)	20 [20 - 20]	20 [20 - 20]	20 [20 - 25]	0.088
FiO_2_ (%)	30 [30 - 38]	30 [30 - 30]	30 [30 - 60]	0.736
Respiratory rate (inspirations/min.)	10 [10 - 10]	10 [10 - 10]	10 [10 - 10]	0.543
Post-ECMO blood gas analysis				
pH	7.39 [7.37 - 7.44]	7.39 [7.36 - 7.40]	7.46 [7.40 - 7.51]	0.080
PaO_2_ (mmHg)	61 [52 - 68]	62 [52 - 68]	53 [49 - 65]	0.375
PaCO_2_ (mmHg)	38 [36 - 42]	40 [36 - 44]	36 [33 - 36]	0.048
SBE (mEq/L)	1.3 [-0.6 - 4.2]	1.0 [-0.5 - 4.0]	1.6 [-3.4 - 8.4]	0.921
PaO_2_ < 40mmHg in ECMO#	5 (28)	3 (23)	2 (40)	0.896
Positive blood cultures at admission				
*Candida sp.*	3 (17)	2 (15)	1 (20)	1.000
*Pseudomonas aeruginosa*	2 (11)	1 (8)	1 (20)	0.490
*Klebsiella pneumoniae*	1 (6)	0 (0)	1 (20)	0.278
*Proteus mirabilis*	1 (6)	1 (8)	0 (0)	1.000
Renal replacement therapy				
Continuous	10 (56)	7 (54)	3 (60)	1.000
Intermittent	6 (33)	6 (46)	0 (0)	0.192
Not used	8 (44)	6 (46)	2 (40)	1.000
Nosocomial infections acquired upon arrival at the referral hospital				
PAV	1 (6)	1 (8)	0 (0)	0.900
UTI	1 (6)	0 (0)	1 (20)	0.278
Catheter infection	0 (0)	0 (0)	0 (0)	1.000

ECMO - extracorporeal membrane oxygenation; FiO_2_ - fraction of inspired oxygen; PSV - pressure-supported ventilation; PCV - pressure-controlled ventilation; APRV - airway pressure release ventilation; PEEP - positive-end expiratory pressure; PaO_2_ - partial oxygen pressure; PaCO_2_ - partial carbon dioxide pressure; SBE - standard base excess; VAP - ventilator-associated pneumonia; UTI - urinary tract infection.

* Value of comparison between survivors and nonsurvivors;

# persistent for more than 6 hours. The results are expressed as the number (%) or the median [interquartile 25 - 75].

**Table 5:** Results of transportation

Characteristics	All patients (n = 18)	Survivors (n = 13)	Nonsurvivors (N = 5)	p value*
Hospital discharge	13 (72)	13 (100)	0 (0)	---
ICU discharge	13 (72)	13 (100)	0 (0)	---
ECMO weaning	15 (84)	13 (100)	2 (40)	---
MV weaning	13 (72)	13 (100)	0 (0)	---
Tracheostomized	5 (28)	5 (38)	0 (0)	---
ECMO time (days)	6 [4-9]	5 [4-9]	6 [3-10]	1.000
MV time (days)	7 [6-18]	7 [6-12]	6 [5-20]	0.580
ICU time (days)	15 [10-21]	16 [12-21]	6 [3-20]	0.289
Length of hospitalization (days)	25 [14-50]	28 [24-50]	6 [3-20]	0.084
Oxygen dependence after discharge	0 (0)	0 (0)	0 (0)	---
Need for dialysis after discharge	1 (6)	1 (8)	0 (0)	---
Cause of death				
Brain death† ‡	---	---	2 (40)	---
Multiple organ dysfunction syndrome	---	---	2 (40)	---
Irreversible pulmonary fibrosis¶	---	---	1 (20)	---

ICU - intensive care unit; ECMO - extracorporeal membrane oxygenation; MV - mechanical ventilation.

* Value of comparison between survivors and nonsurvivors;

† patients with support withdrawal;

‡ one patient with extensive brain hemorrhage and one patient with diffuse ischemia and secondary bleeding;

¶ irreversible fibrosis characterized by the complete absence of vascular scaffold in open lung biopsy after 60 days of support with extracorporeal membrane oxygenation and without signs of improvement. The results are expressed as number (%) or median [interquartile 25-75].

## DISCUSSION

In this case series of 18 severe patients transported to specialized centers with ECMO support in São Paulo, the rate of complications was low, and hospital survival was 72%. Of the patients who were discharged from the hospital, only one needed renal replacement therapy, and none required home oxygen therapy.

Fewer than 2% of the patients admitted to the intensive care unit (ICU) suffered from severe respiratory failure. Of these, fewer than 0.5% were refractory to protective mechanical ventilation and salvage therapy for hypoxemia and severe hypercapnia^(23)^ and sometimes required ECMO support. The low rate of very severe patients limits the ability to maintain a team to perform ECMO support in all ICUs. Therefore, in developed countries, transport with installed ECMO support was used to reduce the risk of transportation to specialized centers, and the patient survival rate was 62% (95%CI 57 - 68%).^(4,15)^ In our series, hospital survival was 72% (95%CI 49 - 88%), in agreement with the data reported in the literature.^(15)^

These results are attributed to two main causes. The first is the use of more rigorous inclusion and exclusion criteria, which resulted in restricting the use of ECMO to highly selected patients because ECMO support seems to have a survival benefit with improved quality of life for patients with few comorbidities and few acute dysfunctions.^(9,24)^ In addition, the application of rescue therapy, such as the use of the prone position before ECMO, is essential whenever possible because this therapy is inexpensive and there is strong evidence that its use improves patient survival.^(25)^ Second, the use of ECMO support can be optimized by providing adequate training and experience to the multidisciplinary team^(18)^ and by the involvement of professionals who possess comprehensive knowledge of emergency care and possible complications during ECMO support.^(26-28)^

In our study, the comparison of survivors and nonsurvivors should be considered preliminary because of the small sample size. However, certain factors should be considered. The initial tidal volume of the patients who died was lower than that of those who survived, suggesting greater severity of lung injuries and poorer lung compliance in the former. The Simplified Acute Physiology Score 3 (SAPS 3) did not differ in the two groups, and the RESP score,^(29)^ which was used in decision-making, was higher in survivors. Although the RESP score was developed as a means of predicting patient survival under ECMO support, other scores that were developed to predict patient survival better address other organic functions and may therefore be more accurate.^(30)^ The Survival After Veno-Arterial ECMO Score (SAVE score) was described, but the effects of using this score were not analyzed because it was used in only one case.

Another relevant factor in our sample of nonsurvivors was that the partial pressure decrease in carbon dioxide (PaCO_2_) from pre- to post-ECMO was critical. This characteristic is known to be related to higher patient mortality in ECMO.^(31)^ This factor may have contributed to the deaths of two patients who progressed to brain death while in the ICU. This outcome alerted us to the importance of the careful initiation of extracorporeal ventilation, especially in hypercapnic patients with gas/blood flow < 1, to ensure a smaller initial decrease in PaCO_2_.

The most serious problems that arose during transport were addressed as follows. (1) Energy failure was avoided by using a hand pump for one patient and by turning off the warning lights for another patient, and the ambulance power inverter was dedicated to the operation of the pump. (2) Only decreases in oxygen saturation < 85% and > 70% were observed. These dessaturations occurred because of the severity of lung injury, associated with a cardiac output. Severe hypoxemia may occur during the acute phase of respiratory support and sometimes needs to be tolerated by the team;^(32)^ although this complication may not directly affect survival or cognitive outcome, it indicates the severity of the patient's condition.^(33)^

Although the sample described in this study does not provide new data, it represents the first case series of patients transported in ECMO in Brazil. However, the results of this study should be viewed with caution for several reasons. First, because the sample size was small, it was not possible to perform a multivariate analysis. Second, the results of the analyses are preliminary and should not be used to change procedures at the bedside. Third, generalization of the results reported here to other centers should be made with caution because the number of ECMO support cases per year was low (5 - 10). Fourth, the indications were restricted to a small subset of patients.

Under certain conditions, ECMO can be an effective and cost-effective therapy. The results of this case series demonstrate that this approach can be effective when restrictive indications are followed, adequate intensive care is provided to avoid complications during hospitalization, and the staff involved in patient care are continuously trained to enable them to treat life-threatening complications that may occur during ECMO support. In our opinion, this can only be achieved in a few centers while maintaining the cost-effectiveness of therapy.

## CONCLUSIONS

Transport of severely ill patients with extracorporeal respiratory support in a Brazilian state was feasible and did not result in severe complications. Despite the small sample size, patient survival to hospital admission was similar to that reported in the literature.
