# Supplementary material for: Characterization of patients transported with extracorporeal respiratory and/or cardiovascular support in the State of São Paulo, Brazil
Source: Rev Bras Ter Intensiva. 2018;30(3):317–26. doi: 10.5935/0103-507X.20180052 (PMC6180471; doi:10.5935/0103-507X.20180052)
Supplement: Supplementary file 1 [file rbti-30-03-0317-suppl1.pdf]

Characterization of patients transported with extracorporeal respiratory and/or cardiovascular support in the State of São Paulo, Brazil

Caracterização de pacientes transportados com suporte respiratório e/ou cardiovascular extracorpóreo no Estado de São Paulo - Brasil

Ho Yeh Li[1], Pedro Vitale Mendes[1,4], Livia Maria Garcia Melro[1,2], Daniel Joelsons[1], Bruno Adler Maccagnan Pinheiro Besen[1,4], Eduardo Leite Viera Costa[1,3], Adriana Sayuri Hirota[1], Edzangela Vasconcelos Santos Barbosa[1], Flavia Krepel Foronda[1], Luciano Cesar Pontes Azevedo[1,3], Thiago Gomes Romano[1,4], Marcelo Park[1]

METHODS

Indication criteria for the use of extracorporeal membrane oxygenation:

- Mechanical ventilation and positive end expiratory pressure ≥10cmH_2O.
- Diffuse infiltrate.
- P/F ratio < 80 with fraction of inspired oxygen > 80%.
- pH < 7.20 due to persistent hypercapnia with mild metabolic acidosis.
- Previous salvage therapy.

Contraindication criteria for the use of extracorporeal membrane oxygenation:

- Dying patient.
- Body mass index > 35.
- Comma without sedatives after cardiorespiratory arrest.
- Patients without central venous access.
- Mechanical ventilation for > 7 days.
- Age > 60 years
- Pre-intensive care unit hospitalization for > 7 days.
- Need for vasopressor > 0.5mcg/kg/min of adrenergic equivalent.
- Dialysis with worsening of metabolic acidosis.
- Any chronic disease with KPS < 70.
- Bridge to transplantation.
- Isolation of multidrug-resistant microorganism.
- Prediction of the impossibility of returning to usual activities.
- Contraindication to the use of anticoagulation.
- Previous organ transplantation.
- AIDS with treatment failure.
- Any neoplasm.
- Hemoglobinopathy.
- Chronic disease (rheumatologic or hematologic, among others)

RESULTS

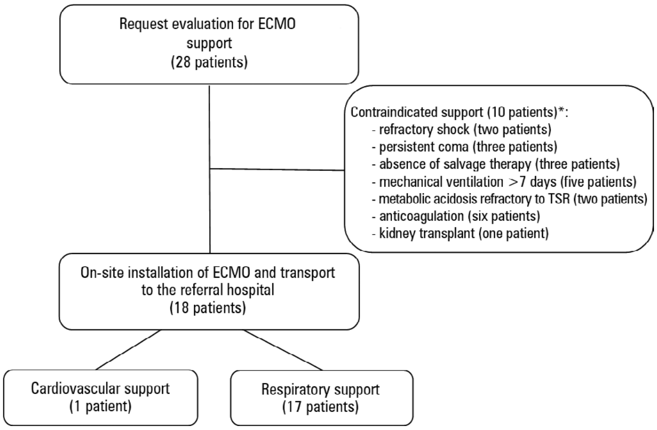

Figure 1S - Distribution of the patients eligible for transport using extracorporeal membrane oxygenation support. ECMO - extracorporeal membrane oxygenation; RRT - renal replacement therapy. * The sum exceeds ten patients because some patients had more than one contraindication.

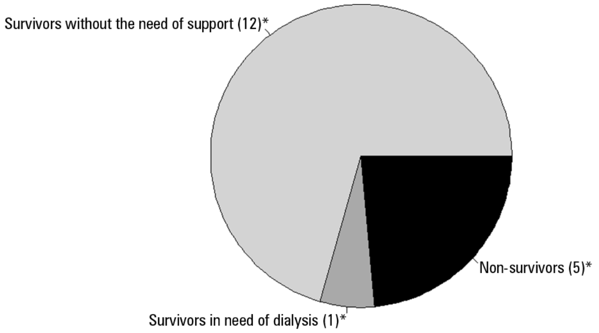

Figure 2S - Survival to hospital admission. * The number of patients per category is shown in parentheses.

Table 1S - Individual data of patients transported with extracorporeal membrane oxygenation support

Number	Gender	Age	Survival	Distance (km)	Mission time (minutes)	RESP/SAVE score	Survival by the RESP/SAVE score (%)	SAPS 3	SOFA	Diagnosis	LIS	P/F ratio (mmHg)	pH	$PaCO_2$ (mmHg)
1	F	14	Yes	1.0	360	-4	30	105	18	Alveolar hemorrhage and SLE	4.00	32	7.36	45
2	M	27	No	40.0	300	-3	35	118	18	Necrotizing pneumonia	4.00	33	7.06	142
3	F	48	Yes	40.0	270	2	60	84	10	Nosocomial pneumonia	3.00	60	7.35	40
4	M	16	No	23.2	420	-7	15	57	7	Aspiration pneumonia	3.00	45	7.34	48
5	F	31	No	15.0	345	-2	40	80	13	H3N2 Influenza A virus	3.70	50	7.00	90
6	M	31	Yes	163.0	660	-2	40	84	13	Varicella zoster virus	4.00	39	7.51	39
7	F	28	Yes	54.2	300	6	85	60	8	Respiratory syncytial virus	4.00	65	7.09	107
8	M	29	No	97.0	435	-1	45	73	13	*Pneumocystis jirovecii*	4.00	60	7.10	90
9	F	59	No	37.0	480	0	50	95	9	H1N1 Influenza A virus	3.75	61	7.39	49
10	M	30	Yes	58.0	420	4	72	53	14	H1N1 Influenza A virus	2.75	62	7.33	40
11	F	44	Yes	90.0	440	4	72	93	16	H1N1 Influenza A virus	4.00	37	7.27	52
12	M	28	Yes	10.0	420	0	50	85	18	Leptospirosis	3.75	24	7.05	73
13	F	25	Yes	37.0	210	1	55	88	14	Leptospirosis	3.50	43	7.32	69
14	M	15	Yes	90.0	360	1	55	86	18	Leptospirosis	3.00	40	6.96	146
15	F	41	Yes	16.0	240	3	68	80	7	Coronavirus	3.25	64	7.27	53
16	F	25	Yes	12.4	330	-1	40	66	7	Pelvic septic thrombophlebitis	3.00	131	7.08	146
17	F	13	Yes	101.0	360	2	60	65	12	Influenza B virus	3.75	74	7.21	80
18	F	29	Yes	2.5	450	0	46[#]	101	14	Epstein-Barr Virus	0.00	175	7.55	42

RESP score - Respiratory ECMO Survival Prediction Score; SAVE score - Survival After Veno-Arterial-ECMO Score; SAPS - Simplified Acute Physiology Score; SOFA - Sequential Organ Failure Assessment Score; LIS - Lung Injury Score or Murray score; P/F ratio - PaO_2 to FiO_2 ratio; $PaCO_2$ - partial pressure of carbon dioxide; F - female; SLE - systemic lupus erythematosus; M - male. [#] Patient with Epstein-Barr virus myocarditis and the only one with a veno-arterial configuration. The data were obtained at the initiation of salvage therapy.
